# High gene flow between alternative morphs and the evolutionary persistence of facultative paedomorphosis

**DOI:** 10.1038/srep32046

**Published:** 2016-08-18

**Authors:** Neus Oromi, Johan Michaux, Mathieu Denoël

**Affiliations:** 1 Laboratory of Fish and Amphibian Ethology, Behavioural Biology Unit, Freshwater and Oceanic Science Unit of Research (FOCUS), University of Liège, 22 Quai van Beneden, 4020 Liège, Belgium; 2 Conservation Genetics, University of Liège, Institute of Botany (Bat. 22), 2 Chemin de la Vallée, 4000 Liège, Belgium

## Abstract

Paedomorphosis and metamorphosis are two major developmental processes that characterize the evolution of complex life cycles in many lineages. Whereas these processes were fixed in some taxa, they remained facultative in others, with alternative phenotypes expressed in the same populations. From a genetic perspective, it is still unknown whether such phenotypes form a single population or whether they show some patterns of isolation in syntopy. This has deep implications for understanding the evolution of the phenotypes, i.e. towards their persistence or their fixation and speciation. Newts and salamanders are excellent models to test this hypothesis because they exhibit both developmental processes in their populations: the aquatic paedomorphs retain gills, whereas the metamorphs are able to colonize land. Using microsatellite data of coexisting paedomorphic and metamorphic palmate newts (*Lissotriton helveticus*), we found that they formed a panmictic population, which evidences sexual compatibility between the two phenotypes. The high gene flow could be understood as an adaptation to unstable habitats in which phenotypic plasticity is favored over the fixation of developmental alternatives. This makes then possible the persistence of a polyphenism: only metamorphosis could be maintained in case of occasional drying whereas paedomorphosis could offer specific advantages in organisms remaining in water.

Polymorphisms, the presence of discrete intraspecific variation, are widespread in a large variety of organisms^1^. The alternative phenotypes can be the result from the expression of one genotype under different environments (i.e., a reaction norm), several genotypes reacting differently to an environmental threshold value (i.e., a genetic polymorphism), or from a genotype expressing different traits depending on their own phenotypic transitions during a lifetime (condition-dependent or status-dependent switch)^2^. In response to environmental pressures, different genotypes with contrasted adaptations could become isolated through sympatric speciation processes^3^,^4^. Some radiations, such as in cichlids, are then thought to have been favored by the intermediate steps of polymorphism^5^. Consequently, it is not surprising that several examples of polymorphisms exhibiting varying degrees of sexual isolation have been described^6^,^7^. However, there is now also increasing evidence that alternative morphs can be maintained within diverging species in radiating clades^8^. In particular, polyphenisms, i.e. environmentally-cued polymorphisms, may even persist over evolutionary time and then allow survival in changing environments^9^. Understanding the patterns of diversity and the processes that generate and maintain them is essential to figure out the possible evolution of phenotypes and the meaning of their existence^9^.

Phenotypic variation can be achieved through heterochronic processes, i.e. changes in the rate or timing of developmental events relative to the same events in the ancestors^10^,^11^. Because alteration of developmental pathways does not necessarily require large genetic changes, it is believed that heterochronies play major roles in micro and macro-evolution^12^. Specifically, when affecting metamorphosis, a widespread transition process between life stages, heterochronies allow specialization on new resources and their exploitation^13^,^14^. At a broader extent, alterations of such developmental patterns and thus the evolution of alternative phenotypes can give rise to the rich diversity of simple and complex life cycles found in metazoans^15^. A famous example of heterochrony is paedomorphosis in caudate amphibians, in which aquatic gilled larvae acquire sexual maturity without metamorphosing into a land-adapted organism^10^. Complex life cycles in amphibians have been highly altered during their evolution with species and even families becoming (1) obligate paedomorphs (i.e., permanently aquatic larvae able of reproduction), (2) obligate metamorphs (i.e., either bi-phasic with metamorphosis or fully terrestrial, skipping the larval stage), and (3) facultative paedomorphs (i.e., with two phenotypes–the paedomorphs and the metamorphs–that can coexist in the same reproductive habitat; Fig. 1)^16^. The fixation of the developmental pathways is believed to have resulted from hard selective pressures on the aquatic versus terrestrial environments^17^. For instance, arid terrestrial conditions may have favored the fixation of paedomorphosis in some ambystomatid and plethodontid salamanders^18^,^19^.

Facultative paedomorphic populations exist in a large number of species of newts and salamanders^16^. On one hand, population variation in the propensity to become paedomorphs and artificial selection of paedomorphosis suggest a genetic basis^20^. On the other hand, the development into a paedomorphic or a metamorphic pathway is dependent on environmental pressures, such as the risk of drying^21^. Behavioral laboratory studies suggested the possibility of sexual compatibility between morphs^22^. However, ecological specializations at spatial and temporal scales may affect isolation between morphotypes^23^,^24^. Moreover, alternative phenotypes differ in the expression of secondary sexual traits and this may make them differently attractive^25^. An explanation to the persistence of facultative paedomorphosis in natural populations is that it allows to cope with environmental heterogeneity; each phenotype being adapted to spatially or temporally available resources^26^,^27^. On one hand, the two phenotypes are specialized on foraging on alternative prey and exploiting different micro-habitats^16^. On the other hand, paedomorphs could avoid the cost of metamorphosis and gain in maturing early by progenesis, whereas metamorphs can survive catastrophes such as drying by dispersing to new habitats^28^.

In this context, the aim of this study was to test two alternative scenarios of the evolution of facultative paedomorphosis. These hypotheses are based on the existence or absence of genetic isolation between the two phenotypes. First, the behavioral specializations of each morph could result in their isolation. Second, the absence of sexual isolation could allow the species to subsist in a temporary environment. Our hypothesis is that it is the second scenario which is the most likely one because catastrophes lead to the extinction of paedomorphs in natural populations^29^. To test this hypothesis, we determined the gene flow between the two alternative phenotypes in using the palmate newt (*Lissotriton helveticus*) as a model species.

## Results

MICRO-CHECKER software did not detect the presence of null alleles at any locus. The ten microsatellite markers were polymorphic in the global population and the number of alleles per locus ranged from 3 for US4 to 8 for LH1, with an average of 5.3 (5.2 in metamorphs and 4.3 in paedomorphs). A total of 53 different alleles were identified: 42 were present in the individuals of the two phenotypes (common alleles), whereas 11 were found only in one of the two phenotypes. The number of private alleles was higher in the metamorphs (i.e., 10) than in the paedomorphs (i.e., 1) and there were significant differences between morphs (Wilcoxon signed-rank test Z = −2.264, P = 0.024). H_E_ ranged from 0.27 to 0.76 and was lower than H_O_ in all cases (with the exception of LH17; Table 1). Significant deviations of Hardy-Weinberg equilibrium were observed in 7 of the 10 markers caused by heterozygote excess (Table 1). After Bonferroni correction for multiple tests, a significant deviation from genotypic linkage equilibrium was found in 6 of the total 90 locus pair tested (Supplementary Table S1). The low deviation from linkage equilibrium observed could be interpreted as a relaxed population structure. The average AR (±s.d.) was 4.56 ± 1.57 for the global population and was significantly lower in the paedomorphs (4.20 ± 1.38) than in the metamorphs (4.91 ± 1.76) (Wilcoxon signed-rank test Z = −2.371, P = 0.018). There was not evidence for non-neutrality of the microsatellite loci based on simulation test using F_ST_-outlier detection implemented in LOSITAN (Supplementary Table S2).

The fixation indexes (F_IS_) were low and negative in all loci in the global population and also both in the metamorphs (overall = −0.310) and paedomorphs (overall = −0.307). Genetic differences between phenotypes (sub-populations) measured by F_ST_ for each locus separately were low, ranging from −0.011 to 0.004 (Table 2), and did not statistically differ (P = 0.81).

**Figure 2:**
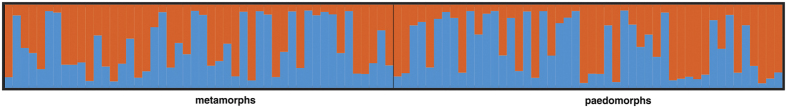
Bayesian population assignment test of paedomorphic and metamorphic palmate newts (Larzac, France). Each vertical bar in the Y-axis represents an individual, and the color composition displays the probability to belong to one of the clusters defined by STRUCTURE (CLUMPAK plot). The black vertical line delineates the pre-defined populations.

Analysis of STRUCTURE, considering Evanno method, identified a most probably clustering value of K = 2 (Supplementary Fig. S1). However, Evanno method cannot find the best K if K = 1 and it is necessary to use other information^30^. In fact, the clustering analysis (Clumpak plot, Fig. 2) and the information provided by STRUCTURE (the value of α = L(K) and individual assignment patterns) showed that all individuals were admixed and could not be assigned to one of the two Ks. The most probable number of clusters for our dataset appears therefore to be K = 1. These results were validated by the AMOVA analysis, which did not show any significant population structure. The observed variation among groups (−0.37%) was insignificant and total variation was found within the groups (100.37%).

## Discussion

The analysis of microsatellite data from the two alternative newt phenotypes demonstrated a lack of genetic divergence between them. To our knowledge, this is the first molecular study evidencing that coexisting paedomorphic and metamorphic newts can form a panmictic population. More broadly, these results are in favor of the evolutionary scenario that predicts the persistence of facultative paedomorphosis as a polyphenism and support recent evidence found in other groups of metazoans that polymorphisms can also be maintained in natural populations rather than being necessarily intermediate steps of speciation^9^,^31^,^32^.

The proximate causes of life history traits variation may be genetic differentiation or phenotypic plasticity or a combination of both^33^. In our study, we found a high gene flow and a lack of neutral genetic structure differentiation between the two phenotypes of *L. helveticus* breeding in the same reproductive habitat. The coexistence of the two phenotypes can be understood as developmental plasticity, which means the ability of an individual to react to environmental input with a change in form^1^. However, the existence of a high gene flow does not mean the absence of alleles favoring the expression of each phenotype. The current localized distribution of paedomorphs is indeed in favor of genetic clusters allowing their expression in palmate newts^34^. In other salamanders, as in ambystomatids, the metamorphic timing is regulated by quantitative trait loci associated with the evolution of paedomorphosis^18^. In the ambystomatids, such as the axolotl, an adaptive pleiotropic basis is suggested, which means that there could be a pathway for the evolution of novel paedomorphic species from metamorphic ancestors via selection of thyroid hormone-response alleles that delay metamorphic timing^35^,^36^,^37^. The implication of thyroid hormones in metamorphosis is not only relevant for amphibians, but at a broader extent to the chordates, therefore indicating the common roots of metamorphosis and its importance in the evolution of organisms^38^.

The selective mechanisms that can promote the evolution of paedomorphosis are complex and vary between species. The existence of one or both phenotypes depends on the costs and benefits gathered by each morph in their respective habitat and according to their mode of life^16^,^27^. Paedomorphs are particularly adapted to aquatic conditions, whereas metamorphs can disperse on land and survive in case of pond drying. Strong environmental conditions can promote isolation or speciation by the disappearance of the other phenotype. In species such as ambystomatids and plethodontids, the long-term changes in aridity patterns are thought to have favored the paedomorphs and speciation in isolated wetlands surrounded by hostile lands^18^,^19^. In contrast, in pond-breeding newts, such as the smooth, palmate or red-spotted newts, the occasional drying of ponds would have prevented the fixation of paedomorphosis and then favored the absence of sexual or ecological isolation between morphs. Because of its polyphenic nature, facultative paedomorphosis can persist even after the disappearance of paedomorphs after pond drying as the metamorphs can recolonize ponds and give birth to progeny becoming paedomorphs^29^. However, in some instances, populations can become more isolated and exposed to increased fragility to disturbance^39^. The connectivity between ponds is thus a key feature to explain the persistence of intra-specific diversity of developmental traits such as paedomorphosis and metamorphosis^34^. In contrast, some other newt species, such as the alpine newt, live in permanent and isolated lakes^28^. It is likely that the evolutionary scenario would be different than in temporary ponds^28^. The stability offered by the aquatic environment and its heterogeneous structure favors there a prolonged aquatic life and in some conditions paedomorphosis^16^. Paedomorphs can exploit vacant niches in deep waters but may also be constrained by a low growth rate in cold waters^40^. Because of isolation, and possibly the harshness of some terrestrial landscapes, dispersal is also less likely than in networks of ponds. Yet, even in such lake systems, paedomorphosis has a low resistance to disturbance. It is exemplified by fish introductions that makes paedomorphosis extirpated from lakes whereas metamorphosis could be maintained^41^. There is unfortunately not yet documented cases of resilience of paedomorphosis after fish removal in such deep lakes. It is possibly due to a too long period of selection against paedomorphosis, which could have reduced the likelihood of an individual to become a paedomorph (see also^20^). Genetic studies at varied scales and environments would be a very exciting perspective of the present work.

Variation among individuals in reproductive success, population bottlenecks, and local extinction contribute to the development of fine-scale genetic subdivision by increasing the rate of stochastic fluctuation in allele frequencies at a given location^42^,^43^. In a metapopulation, if most gene flow comes from nearby populations experiencing similar natural selection, it could facilitate adaptation by supplying additive genetic variation, creating heterosis and decreasing inbreeding depression^44^. In fact, in our study, H_O_ was higher than H_E_ in all loci with a significant HW disequilibrium (i.e., in 7 of the 10 loci). In a metapopulation formed by a network of ponds in which the number of individuals fluctuate over time, random changes in allele frequencies can lead to increased heterozygosity following a population decline^45^. These patterns are typical for the Larzac plateau because population crashes can be frequent (i.e., likely to occur during each newt generation^26^) and ponds are connected, thus offering the potential of fast colonization by metamorphs from nearby sites^26^,^29^,^34^. Such patterns also occurred in the study population where newts suffered much from summer drought, causing low population sizes, particularly in paedomorphs in the years preceding the genetic analysis^46^. On the other hand, selection against inbreeding, that means through reproduction with the most genetically different individuals or through differences of survival between homozygotes and heterozygotes, could also explain the excess of heterozygotes^47^. This is particularly expected in amphibians such as the palmate newt because of a low survival at the pre-hatching and larval stages^48^.

In conclusion, the evolution of polyphenisms such as facultative paedomorphosis depends on the existence or absence of behavioral or ecological isolation between alternative phenotypes. In our study, we have evidenced the coexistence of two phenotypes in the same habitat in an evolutionary scenario that predicts the persistence of facultative paedomorphosis as a polyphenism. The lack of inbreeding and the presence of a heterozygosity excess could be understood as a strategy which is adaptive under environmental change, with each phenotype displaying higher performance in contrasted habitats but constrained by the temporal heterogeneity of their habitat.

## Methods

### Population sampling

We captured 96 palmate newts (48 metamorphs and 48 paedomorphs with 24 individuals of each sex) in the same pond (Le Coulet “North-East”, Larzac Plateau, France, 43.820°N, 3.540°E). The adulthood of each individual was established by the presence of a developed cloaca, i.e. well swollen in both sexes and with groves in females^22^. This trait is only present in adults^22^ and as it was present in all the studied individuals, this study was well based only on adult phenotypes, i.e. paedomorphs and metamorphs. The width of the cloaca had a mean ± s.e. (minimum - maximum) of 4.99 ± 0.06 mm (4.20–5.35 mm) and 3.71 ± 0.08 mm (3.03–4.40 mm) in metamorphic and paedomorphic males, respectively and 2.74 ± 0.06 mm (2.19–3.41 mm) and 1.92 ± 0.04 mm (1.54–2.14 mm) in metamorphic and paedomorphic females, respectively (*n* = 24 for each group). The males had also several secondary sexual traits, such as a tail filament. Immature newts never show these sexual traits^22^. The individuals were also collected during their reproductive period, as confirmed by the eggs laid by the paedomorphic and metamorphic females and the courtship display exhibited by the paedomorphic and metamorphic males when placed in aquaria. Newts were anesthetized in phenoxyethanol (0.5%) before sampling a small piece of tissue (<3 mm^2^) from the tail fin. This part of the tail regenerates fast and such sampling has no detrimental effects on newts^49^. The tissue samples were stored individually in 90% ethanol until extraction. All newts were released in their native pond after the study in accordance with the collecting permit.

### DNA extraction and genotyping

DNA was extracted from the tail tip using Qiagen DNeasy_Blood & Tissue Kits according to the protocol. Genetic analysis was based on the study of ten microsatellite loci (Lh1, Lh2, Lh13, Lh14, Lh16, Lh17, Lh19, Lh44, Us4, and Us9) previously described by Drechsler *et al*.^50^ (Supplementary Table S3). Microsatellite loci were amplified using 2 μL of the extracted DNA (20 ng) and polymerase chain reaction (PCR) using Multiplex PCR Kit (Qiagen). Briefly, the oligo forward of each set of primers was labelled by one of the four 6-FAM, TET, ATTO 565 and NED fluorochromes. PCR amplification was performed by first denaturing at 94 °C for 5 min, followed by 34 cycles of 1 min at 94 °C, 1 min at 60 °C, and 1 min at 72 °C. A final extension step was carried out by incubating samples for 10 min at 72 °C. Amplified products were analysed by capillary electrophoresis on an ABI 3130xl Genetic Analyser (Applied Biosystems). Allele sizes were determined using GeneScan-500 LIZ standard marker (Applied Biosystems). Genotype calls were obtained using GeneMapper v.3.7 (Applied Biosystems).

### Data analysis

All analyses were based on a sample size of 96 individuals (48 paedomorphs and 48 metamorphs). Potential genotyping errors such as presence of null alleles were analysed using MICRO-CHECKER v. 2.2.3^51^. Allele and genotype frequencies, number of alleles per locus (N_A_), and expected (H_E_) and observed (H_O_) heterozygosity and allelic richness (AR) were obtained for each morph (i.e., for the paedomorphs and the metamorphs) using FSTAT v. 2.9.3^52^. Genotype frequencies were tested for conformity to Hardy**–**Weinberg equilibrium by GENEPOP v.4.2^53^, using the Markov chain permutations (1000 dememorizations, 100 batches, 1000 iterations per batch) according to the algorithm of Guo & Thompson^54^. This package was also used to evaluate the marker-to-marker genotypic disequilibrium adjusting for Bonferroni correction. Microsatellite neutrality was tested using LOSITAN, which is a selection-detection workbench based on a well-evaluated F_ST_-outlier detection method^55^.

The overall and pairwise F_ST_ values were calculated to analyze the differentiation among phenotypes in allele frequencies, and F_IS_ to estimate the fixation index using FSTAT v2.9.3^52^. ARLEQUIN v3.1^56^ software was used to test pairwise F_ST_ significance by 10,000 permutations, and the genetic variability was evaluated within and among the two phenotypes with a molecular analysis of variance (AMOVA). STRUCTURE v2.2^57^ was used to infer the number of possible genetic clusters (K) in our dataset, using the admixture and correlated allele frequencies model. 20 independent runs for K = 1–5 were tested (100,000 iterations) with a burn-in period of 50,000.

STRUCTURE HARVESTER v0.6.94 was used to infer the K using the Evanno method^58^. Evanno *et al*.^30^ suggested that the most probable K was inferred from the modal value of rate of change of the LnP(D) value between successive runs (ΔK). Structure plots were created with Clumpak, which compares all runs at each value of K to identify optimal clustering scenarios^59^.

The significance of differences in allelic richness and number of private alleles between two phenotypes was tested using the Wilcoxon signed-ranks test by STATISTICA 10. This test is appropriate because homologous loci were used in the two phenotypese^60^. All tests were two-tailed with α = 0.05.

### Ethical note

The capture permit of newts was issued by DREAL Languedoc-Roussillon, following approval by the Conseil National de la Protection de la Nature (decree 2013274-0001). All manipulations were carried out in accordance with all relevant guidelines and regulations. They were approved by the animal ethical committee of the institution where was carried out the study, i.e. the University of Liège (authorization 1613). All newts were released in their capture location after sampling, following the recommendation of the capture permit.

## Additional Information

**How to cite this article**: Oromi, N. *et al*. High gene flow between alternative morphs and the evolutionary persistence of facultative paedomorphosis. *Sci. Rep.* **6**, 32046; doi: 10.1038/srep32046 (2016).

## Supplementary Material

Supplementary Information

## Figures and Tables

**Figure 1:**
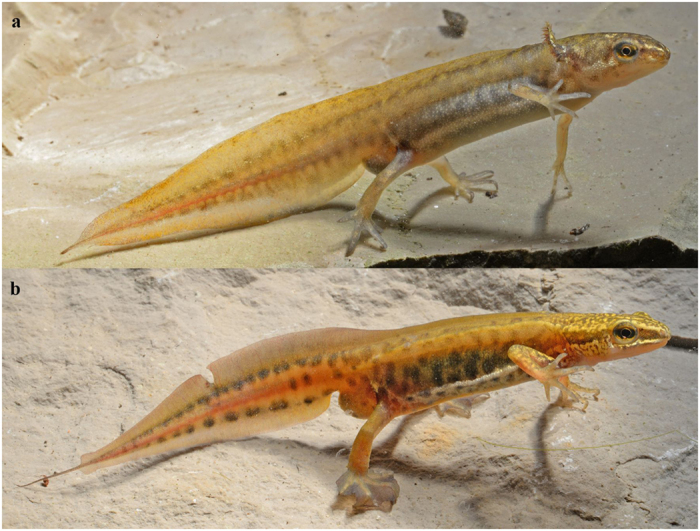
Alternative heterochronic phenotypes in palmate newts. The aquatic paedomorph (**a**) retains gills at the adult stage whereas the metamorph (**b**) is a metamorphosed adult that is adapted for life on land. Photographs by M. Denoël.

**Table 1:** Genetic diversity parameters of coexisting paedomorphic and metamorphic palmate newts (Larzac, France).

Locus	Metamorphs				Paedomorphs				Global population			
	N_A_	He	Ho	AR	N_A_	He	Ho	AR	N_A_	He	Ho	AR
LH1	8 (1)	0.73	1.00*	8.00	7	0.75	1.00**	7.00	8	0.74	1.00**	7.47
US9	8 (3)	0.71	0.94**	6.83	5	0.70	0.98**	4.92	8	0.70	0.95**	5.80
LH44	6 (2)	0.67	0.85	5.72	5 (1)	0.64	0.85*	4.72	7	0.66	0.82**	5.48
LH16	5	0.65	0.98**	5.00	5	0.69	1.00**	5.00	5	0.67	0.99**	4.99
LH19	7 (2)	0.71	0.98**	6.33	5	0.70	1.00**	4.92	7	0.71	0.97**	5.67
LH2	4 (1)	0.52	1.00**	3.48	3	0.51	1.00**	2.72	4	0.52	0.97**	2.96
LH13	4	0.43	0.50	4.00	4	0.45	0.77	4.00	4	0.44	0.47	3.98
LH14	4 (1)	0.44	0.36	3.97	3	0.38	0.17	2.98	4	0.41	0.26**	3.69
LH17	3	0.29	0.35	2.74	3	0.27	0.67	2.71	3	0.28	0.33	2.59
US4	3	0.37	0.44	3.00	3	0.46	0.53	3.00	3	0.42	0.49	3.00
Overall	5.2	0.55	0.74	4.91	4.2	0.55	0.80	4.20	5.3	0.55	0.73	4.74

N_A_ = number of alleles; (n) number of private alleles; He = Expected Heterozygosity; Ho = Observed Heterozygosity with deviation from Hardy–Weinberg proportions (*P < 0.01; **P < 0.001); AR = Allele Richness.

**Table 2:** Fixation index (Fis) and genetic differentiation (pairwise Fst values) within and between the coexisting phenotypes (M = metamorphs; P = paedomorphs) of palmate newts (Larzac, France).

Locus	F_IS_ M	F_IS_ P	F_IS_ Total	F_ST_
LH1	−0.363	−0.326	−0.344	−0.006
US9	−0.330	−0.362	−0.346	−0.005
LH44	−0.257	−0.239	−0.248	0.004
LH16	−0.498	−0.451	−0.474	−0.003
LH19	−0.373	−0.362	−0.368	−0.004
LH2	−0.918	−0.831	−0.874	−0.001
LH13	−0.174	0.015	−0.075	−0.004
LH14	0.187	0.549	0.359	−0.011
LH17	−0.185	−0.161	−0.174	−0.008
US4	−0.190	−0.170	−0.179	−0.001
Overall	−0.310	−0.307	−0.308	−0.003
