# Supplementary material for: High gene flow between alternative morphs and the evolutionary persistence of facultative paedomorphosis
Source: Sci Rep. 2016 Aug 18;6:32046. doi: 10.1038/srep32046 (PMC4989185; doi:10.1038/srep32046)
Supplement: Supplementary Information [file srep32046-s1.pdf]

Supplementary information for

High gene flow between alternative morphs and the evolutionary persistence of facultative paedomorphosis

Neus Oromi[1], Johan Michaux[2] & Mathieu Denoël[1]

[1] Laboratory of Fish and Amphibian Ethology, Behavioural Biology Unit, Freshwater and Oceanic Science Unit of Research (FOCUS), University of Liège, 22 Quai van Beneden, 4020 Liège, Belgium. [2] Conservation Genetics, University of Liège, Institute of Botany (Bat. 22), 2 Chemin de la Vallée, 4000 Liège, Belgium. Correspondence should be addressed to M.D. (Mathieu.Denoel@ulg.ac.be).

Supplementary Table S1. Genotypic linkage disequilibrium analysis of paedomorphic and metamorphic palmate newts (Larzac, France). The analyses are based on a dataset containing 96 palmate newts (Larzac, France) genotyped for 10 polymorphic microsatellite loci.* Significance after Bonferroni correction.

**Metamorphs**			**Paedomorphs**		
**Locus comparison**		**P-Value**	**Locus comparison**		**P-Value**
LH1	US9	0.00001*	LH1	US9	0.00001*
LH1	LH44	0.00668	LH1	LH44	0.54200
LH1	LH16	0.02780	LH1	LH16	0.19473
LH1	LH19	0.00001*	LH1	LH19	0.00001*
LH1	LH2	0.78790	LH1	LH2	0.18288
LH1	LH13	0.37299	LH1	LH13	0.07603
LH1	LH14	0.47625	LH1	LH14	0.80618
LH1	LH17	0.26725	LH1	LH17	0.00681
LH1	US4	0.88376	LH1	US4	0.15395
LH13	LH14	0.05632	LH13	LH14	0.20202
LH13	LH17	0.02148	LH13	LH17	0.92708
LH13	US4	0.68801	LH13	US4	0.08204
LH14	LH17	0.08026	LH14	LH17	0.15823
LH14	US4	0.39642	LH14	US4	0.07358
LH16	LH19	0.04741	LH16	LH19	0.13952
LH16	LH2	1.0000	LH16	LH2	0.19530
LH16	LH13	0.09685	LH16	LH13	0.87864
LH16	LH14	0.27759	LH16	LH14	0.68029
LH16	LH17	0.54589	LH16	LH17	0.92846
LH16	US4	0.36961	LH16	US4	0.81096
LH17	US4	0.01769	LH17	US4	0.32326
LH19	LH2	0.78371	LH19	LH2	0.15879
LH19	LH13	0.19464	LH19	LH13	0.36023
LH19	LH14	0.12509	LH19	LH14	0.47849
LH19	LH17	0.06822	LH19	LH17	0.13154
LH19	US4	0.49031	LH19	US4	0.51038
LH2	LH13	0.41195	LH2	LH13	0.58546
LH2	LH14	1.0000	LH2	LH14	0.04095
LH2	LH17	0.14491	LH2	LH17	0.44695
LH2	US4	0.44108	LH2	US4	0.83693
LH44	LH16	0.01442	LH44	LH16	0.04722
LH44	LH19	0.02011	LH44	LH19	0.31422
LH44	LH2	0.60460	LH44	LH2	1.0000
LH44	LH13	0.03852	LH44	LH13	0.39847
LH44	LH14	0.43482	LH44	LH14	0.39368
LH44	LH17	0.63641	LH44	LH17	0.60977
LH44	US4	0.07207	LH44	US4	0.76753
US9	LH44	0.09272	US9	LH44	0.34621
US9	LH16	0.01914	US9	LH16	0.11056
US9	LH19	0.00001*	US9	LH19	0.00001*
US9	LH2	0.81490	US9	LH2	0.21107
US9	LH13	0.15077	US9	LH13	0.38387
US9	LH14	0.32994	US9	LH14	0.46460
US9	LH17	0.33258	US9	LH17	0.10715
US9	US4	0.43952	US9	US4	0.50955

Supplementary Table S2. Microsatellite neutrality test using LOSITAN under infinite allele mutation models. The analyses are based on a dataset containing 96 palmate newts (Larzac, France) genotyped for 10 polymorphic microsatellite loci.

Locus	H_E	F_{ST}	P (Simul F_{ST} < sample F_{ST})	Selection Status
LH1	0.7392	-0.0112	0.2069	Neutral
LH13	0.4381	-0.0049	0.5251	Neutral
LH14	0.4031	-0.0071	0.5233	Neutral
LH16	0.6692	-0.0084	0.3843	Neutral
LH17	0.2791	-0.0097	0.4582	Neutral
LH19	0.7057	-0.0074	0.3909	Neutral
LH2	0.5161	-0.0102	0.3980	Neutral
LH44	0.6577	0.0017	0.6099	Neutral
US4	0.4138	-0.0026	0.6074	Neutral
US9	0.7007	-0.0086	0.3518	Neutral

Supplementary Table S3. Dataset containing 96 palmate newts (Larzac, France) genotyped for 10 polymorphic microsatellite loci. I = individual code; S = sex, F = female, M = male; Mo = morph, m = metamorph, p = paedomorph, A1 = allele 1, A2 = allele 2.

I	S	Mo	LH1 A1	LH1 A2	US9 A1	US9 A2	LH44 A1	LH44 A2	LH16 A1	LH16 A2	LH19 A1	LH19 A2	LH2 A1	LH2 A2	LH13 A1	LH13 A2	LH14 A1	LH14 A2	LH17 A1	LH17 A2	US4 A1	US4 A2
1	F	m	175	190	175	190	215	236	118	126	175	190	158	174	182	186	224	228	174	198	184	184
2	M	m	194	197	186	194	223	236	114	120	187	194	158	174	182	182	228	228	174	198	181	184
3	F	m	186	194	186	194	223	236	122	126	187	194	158	174	182	199	217	228	174	198	181	184
4	M	m			186	186	215	236	118	120	187	187	158	174	186	190	213	217	174	198	184	184
5	F	m	173	186	186	190	223	236	114	120	187	190	158	174	182	182	224	228	174	174	184	184
6	M	m	186	194	186	194	223	236	114	120	187	194	158	174	182	199	224	228	174	198	181	184
7	F	m	175	186	175	186	223	236	114	120	175	187	158	174	182	199	228	228	174	174	184	184
8	M	m			175	186	236	236	122	126	175	187	158	174	182	182	228	228	174	174	184	188
9	F	m			175	190	236	236	114	122	175	190	158	174	182	182	224	228	174	174	184	184
10	M	m	186	194	186	190	215	236	114	120	187	194	158	174	182	182	228	228	174	198	184	184
11	F	m	180	186	180	186	223	236	114	120	180	187	158	174	182	182	228	228	174	174	184	184
12	M	m	175	186	175	186	223	236	114	120	175	187	158	174	182	182	224	228	174	174	184	184
13	F	m	194	197	186	194	215	236	114	122	187	194	158	174	182	182	228	228	174	198	184	188
14	M	m			186	194	236	236	114	120	187	194	158	174	182	182	228	228	174	174	184	184
15	F	m	175	186	175	186	223	236	114	120	175	187	158	174	182	182	228	228	174	174	184	184
16	M	m			175	186	223	236	114	120	175	187	158	174	182	182	224	224	174	174	184	188
17	F	m	173	186	186	190	223	236	114	120	187	190	158	174	182	199	228	228	174	174	184	184
18	M	m	173	186	175	186	232	236	114	120	175	187	158	174	182	199	224	228	174	198	184	188
19	F	m			175	186	215	223	114	120	175	187	158	174	182	199	228	228	174	174	184	184
20	M	m	186	190	186	186	215	236	118	120	187	190	158	174	182	182	228	228	174	174	184	184
21	F	m	190	194	190	194	236	236	114	120	190	194	158	174	182	182	228	228	174	174	181	184

22	M	m	175	186	186	194	223	236	114	120	187	194	162	174	182	199	228	228	174	198		
23	F	m	175	186	175	186	223	236	114	120	175	187	158	174	182	199	228	228	174	174	184	188
24	M	m	173	186	186	186	232	236	118	126	175	187	158	174	182	186	224	228	174	198	181	184
25	F	m			186	194			114	120	187	194	158	174	182	190	228	228	174	174	184	184
26	M	m	175	186	175	186	223	236	114	120	175	187	158	174	182	199	224	228	174	198	181	184
27	F	m	175	186	175	186	236	236	114	120	175	187	158	174	182	182	228	228	174	198	184	188
28	M	m			175	194	223	236	114	120	175	194	158	174	182	182	224	224	174	174	184	184
29	F	m	175	186	175	186	223	232	122	126	175	187	158	174	182	199	228	228	174	198	181	184
30	M	m			175	194	215	236	114	120	175	194	158	174	182	182	224	228	174	174	184	184
31	F	m			182	194	223	236	114	120	182	194	158	174	182	182	224	228	174	174	184	184
32	M	m			175	194	215	223	114	120	175	194	158	174	182	186	228	228	174	174	184	184
33	F	m	169	175	168	173	210	221	114	118	168	175	158	174	182	199	228	228	174	198	184	188
34	M	m			175	194	232	236	114	120	175	194	158	174	182	190	224	224	174	174	184	188
35	F	m	175	186	175	186	223	236	114	120	175	187	158	174	182	182	228	228	174	174	181	184
36	M	m	175	186	175	186	223	236	114	120	175	187	158	174	182	199	224	228	174	174	184	184
37	F	m	175	186	175	186	223	236	114	120	175	187	158	174	182	182	228	228	174	174	181	184
38	M	m	175	186	175	186	223	236	114	120	175	187	158	174	182	190	213	228	174	194	184	184
39	F	m	175	186	175	186	215	223	114	120	175	187	158	174	182	182	228	228	174	174	184	184
40	M	m	186	194	175	186	223	232	114	120	175	187	158	174	182	199	228	228	174	174	184	184
41	F	m	175	186	175	186			114	120	175	187										
42	M	m	186	194	175	186	236	236	114	120	175	187	174	198	182	199			174	198	184	188
43	F	m	175	186	175	186	223	236	114	120	175	187	158	174	182	182	217	228	174	174	184	184
44	M	m	175	186	175	186	223	236	114	118	175	187	158	174	182	182	228	228	174	174	184	184
45	F	m			175	186	221	221	114	114	175	182										
46	M	m	186	190	186	190	215	236	120	126	187	190	158	174	182	199	228	228	174	174	184	188
47	F	m	175	194	175	194	215	236	114	120	175	194	158	174	182	182	224	224	174	174	184	188

48	M	m			175	190	215	232	114	120	175	190	158	174	182	186	213	228	174	174	184	184
49	F	p	186	194	186	194	223	236	114	120	187	194	158	174	182	182	228	228	174	174	181	184
50	M	p	186	190	186	190	236	236	118	120	187	190	158	174	182	186	228	228	174	174	184	184
51	F	p			175	194	223	236	114	120	175	194	158	174	182	199	224	224	174	174	184	184
52	M	p	186	194	186	194	232	236	114	126	187	194	158	174	182	182	228	228	174	198	184	188
53	F	p			186	194			114	120	187	194	158	174	186	199	228	228	174	174	184	184
54	M	p			175	186	223	223	114	120	175	187	158	174	182	186	228	228	174	198	181	184
55	F	p	169	186	186	194	223	236	114	120	187	194	174	198	186	199	228	228	174	198	184	184
56	M	p			175	186	223	236	118	120	175	187	158	174	182	182	224	224	174	174	184	188
57	F	p	175	186	175	186	223	236	114	120	175	187	158	174	182	182	228	228	174	174	181	184
58	M	p	186	194	186	194	223	236	114	120	187	194	158	174	182	182	228	228	174	174	184	184
59	F	p	180	194	180	194	223	236	122	126	180	194	158	158	182	182	224	224	174	174	184	188
60	M	p	186	194	186	194	223	236	114	126	187	194	158	174	182	186	228	228	174	174	181	184
61	F	p	169	175	175	186	223	232	120	126	175	187	158	174	182	190	228	228	174	174	181	184
62	M	p	173	190	190	194	223	232	114	122	190	194	158	174	182	186	228	228	174	198	181	184
63	F	p	173	194	194	194	223	236	114	122	187	187	158	174	182	199	228	228	174	198	184	184
64	M	p			186	190	232	236	114	120	187	190	158	174	182	186	228	228	174	174	184	184
65	F	p	175	186	175	186	223	236	114	120	175	187	158	174	182	182	228	228	174	174	184	188
66	M	p	186	190	186	190	236	236	114	122	187	190	158	158	182	199	224	224	174	174	181	184
67	F	p			186	190	236	236	114	122	187	190	158	174	182	182	224	228	174	174	184	184
68	M	p			186	194	223	236	114	120	187	194	158	174	186	199	217	228	174	174	184	188
69	F	p			175	194	215	236	120	126	175	194	158	174	182	182	228	228	174	174	184	184
70	M	p	186	190	186	194	223	236	114	120	187	194	158	174	182	182	224	228	174	198	181	184
71	F	p	173	186	175	186	223	223	114	120	175	187	158	174	182	182	224	228	174	198	181	184
72	M	p	173	186	175	186	215	236	114	120	175	187	158	174	182	182	228	228	174	198	181	184
73	F	p	175	186	175	186	223	223	114	120	175	187	158	174	186	190	228	228	174	174	184	188

74	M	p	186	194	186	186	223	236	114	120	194	194	158	174	182	182	224	224	174	194	184	184
75	F	p			175	194	236	236	114	120	175	194	158	174	182	199	228	228	174	174	184	184
76	M	p	190	194	190	194	223	236	114	120	190	194	158	174	182	199	228	228	174	198	188	188
77	F	p			180	186	236	236	118	120	180	187	158	174	182	182	224	228	174	174	181	184
78	M	p	173	186	175	186	223	236	118	120	175	187	158	174	182	182	224	228	174	198	181	184
79	F	p	175	186	175	186	232	236	114	118	175	187	158	174	182	182	228	228	174	174	184	184
80	M	p	175	186	175	186	223	236	114	120	175	187	158	174	182	182	228	228	174	174	184	184
81	F	p	175	186	175	186	223	236	114	120	175	187	158	174	182	182	224	228	174	174	181	184
82	M	p	175	186	175	186	223	232	118	126	175	187	158	174	182	199	228	228	174	174	184	184
83	F	p	186	190	186	190	223	236	114	120	187	190	158	174	182	182	228	228	174	198	184	188
84	M	p			175	186	215	223	114	126	175	187	158	174	182	182	228	224	174	174	181	184
85	F	p	175	186	175	186	215	236	114	120	175	187	158	174	190	199	228	228	174	198	184	184
86	M	p	175	186	175	186	236	236	114	120	175	187	158	174	182	190	228	228	174	174	184	188
87	F	p	180	186	175	186	223	236	114	120	175	187	158	174	182	186	228	228	174	174	184	184
88	M	p	175	186	175	186	223	236	114	120	175	187	174	174	182	182	224	224	174	174	184	184
89	F	p	175	186	175	186	215	236	114	120	175	187	158	174	182	182	228	228	174	174	184	184
90	M	p	175	186	175	186	223	236	114	120	175	187	158	174	182	182	228	228	174	174	184	188
91	F	p	175	190	175	190	223	228	114	120	175	190	158	174	182	182	228	228	174	174	184	184
92	M	p	175	194	175	194	223	232	118	120	175	194	158	174	182	190	228	228	174	198	184	188
93	F	p			175	186	223	236	114	120	175	187	158	174	182	199	228	228	174	198	184	184
94	M	p	175	186	175	186	223	232	114	120	175	187	158	174	182	182	228	228	174	174	184	188
95	F	p	186	194	186	194	232	236	122	126	187	194							174	174		
96	M	p	175	186	175	186	236	236	118	120	175	187	158	174	182	182	217	217	174	174	184	184

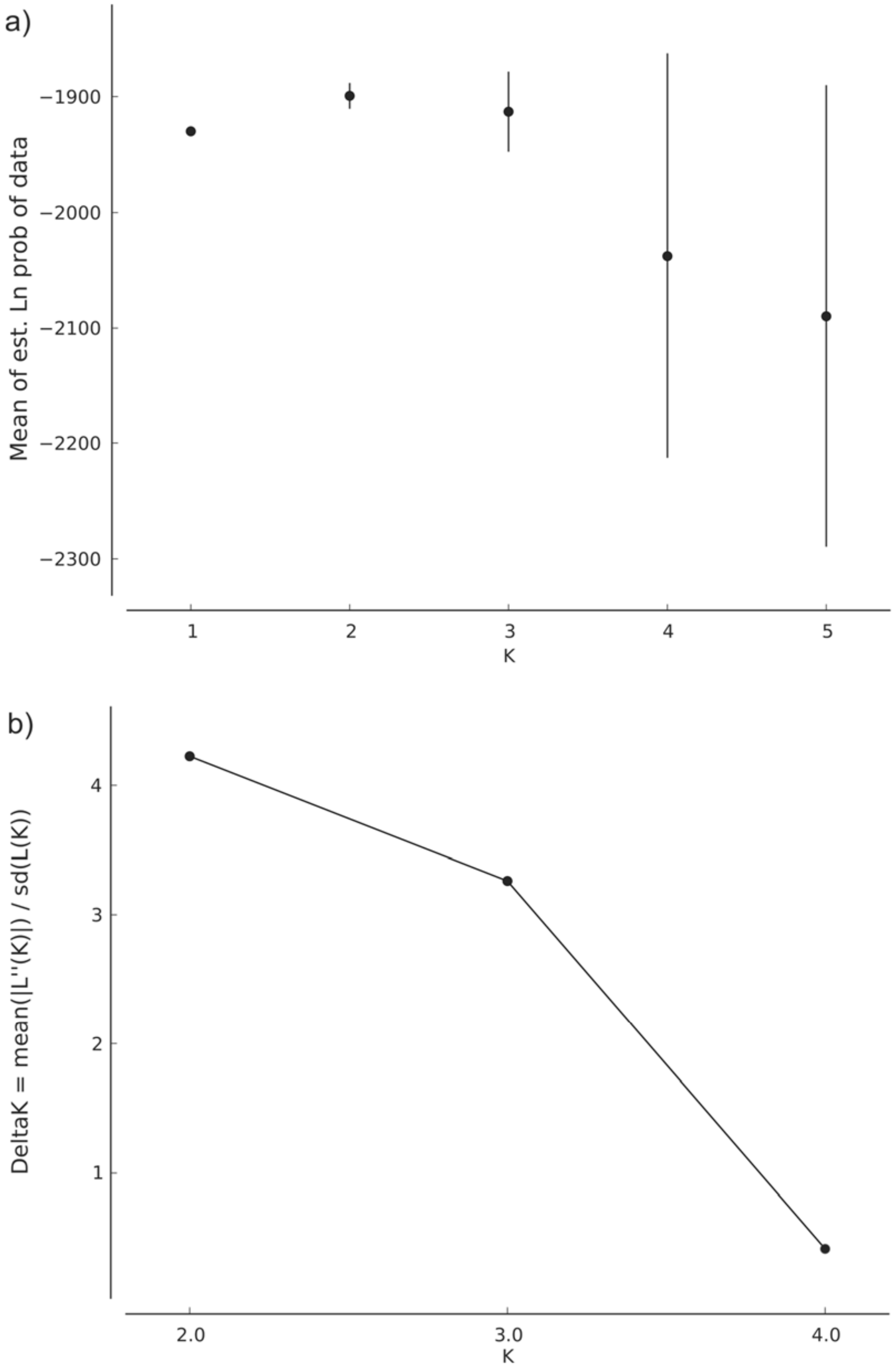

Supplementary Figure S1. Results of STRUCTURE HARVESTER: (a) Plot of mean likelihood L(K) and standard deviation per cluster (K) value and (b) Evanno plot for detecting the number of K groups. The analyses are based on a dataset containing 96 palmate newts (Larzac, France) genotyped for 10 polymorphic microsatellite loci. Delta K is the rate of change of log likelihood.
